# Supplementary material for: Membrane Fouling Controlled by Adjustment of Biological Treatment Parameters in Step-Aerating MBR
Source: Membranes (Basel). 2021 Jul 22;11(8):553. doi: 10.3390/membranes11080553 (PMC8399131; doi:10.3390/membranes11080553)
Supplement: Supplementary file 1 [file membranes-11-00553-s001.zip › Supplementary material.pdf]

membranes

Supplementary Material

Table S1. Specifications of the membrane module.

Membrane configuration	A4 flat sheet (Type H 203, Kubota)
Base of membrane sheet	Polyethylene terephthalate (PET)
Filtration material of membrane sheet	Chlorinated polyethylene (CPE)
Nominal pore size	0.4 μm
Effective membrane area	0.11 m^2
Clean water initial permeate flow	7.2 L/h
